# Photodynamic‐Chemodynamic Cascade Reactions for Efficient Drug Delivery and Enhanced Combination Therapy

**DOI:** 10.1002/advs.202002927

**Published:** 2021-04-08

**Authors:** Sheng Wang, Guocan Yu, Weijing Yang, Zhantong Wang, Orit Jacobson, Rui Tian, Hongzhang Deng, Lisen Lin, Xiaoyuan Chen

**Affiliations:** ^1^ School of Life Sciences Tianjin University Tianjin 300072 China; ^2^ Laboratory of Molecular Imaging and Nanomedicine National Institute of Biomedical Imaging and Bioengineering National Institutes of Health Bethesda MD 20892 USA; ^3^ MOE Key Laboratory for Analytical Science of Food Safety and Biology & Institute of Environmental Analysis and Detection College of Chemistry Fuzhou University Fuzhou 350108 China; ^4^ Departments of Diagnostic Radiology, Chemical and Biomolecular Engineering, and Biomedical Engineering National University of Singapore Singapore 117545 Singapore

**Keywords:** cascade reaction, combination therapy, nanomedicine, reactive oxygen species, triggered drug release

## Abstract

Nanomedicines with photodynamic therapy and reactive oxygen species (ROS)‐triggered drug release capabilities are promising for cancer therapy. However, most of the nanomedicines based on ROS‐responsive nanocarriers still suffer from serious ROS consumption during the triggered drug release process. Herein, a photodynamic‐chemodynamic cascade strategy for the design of drug delivery nanosystem is proposed. A doxorubicin hydrochloride‐loaded ROS‐responsive polymersome (DOX‐RPS) is prepared via the self‐assembly of amphiphilic poly(ethylene glycol)‐poly(linoleic acid) and poly(ethylene glycol)‐(2‐(1‐hexyloxyethyl)‐2‐devinyl pyropheophorbide‐α)‐iron chelate (PEG‐HPPH‐Fe). The RPS can effectively deliver a drug to tumor site through passive targeting effect. Upon laser irradiation, the photosensitizer HPPH can efficiently generate ROS, which further causes in situ oxidation of linoleic acid chain and subsequent RPS structural destruction, permitting triggered drug release. Intriguingly, catalyzed by HPPH‐Fe, ROS will be regenerated from linoleic acid peroxide through a chemodynamic process. Therefore, ROS‐triggered drug release can be achieved without ROS over‐consumption. The in vitro and in vivo results confirmed ROS generation, triggered drug release behavior, and potent antitumor effect of the DOX‐RPS. This photodynamic‐chemodynamic cascade strategy provides a promising approach for enhanced combination therapy.

## Graphical abstract

Photodynamic‐chemodynamic cascade strategy: A reactive oxygen species (ROS)‐responsive polymersome is developed for chemo‐photodynamic combination cancer therapy. The ROS generated by photodynamic effect will cause ROS‐triggered drug release. Furthermore, ROS could be regenerated through a Fenton‐like reaction. Therefore, excessive ROS consumption in the drug release process can be avoided.

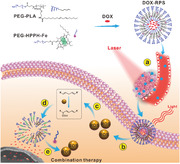

Chemotherapy is still an important approach in cancer treatment. Unfortunately, the conventional chemotherapy with systemic administration often suffers from nonspecific biodistribution of therapeutic drugs, leading to low bioavailability and severe side effects.^[^ ^1^ ^]^ In the past few decades, the development of nanomedicines has shown great promise for drug delivery to address the above‐mentioned drawbacks of systemic chemotherapy through passive and active tumor targeting.^[^ ^2^ ^]^ Particularly, stimuli‐responsive nanomedicines can realize on‐demand drug release, which can further improve bioavailability of therapeutic drugs.^[^ ^3^ ^]^ The design of nanocarriers that can respond to certain stimuli and realize structural destruction is a frequently used approach for the development of stimuli‐responsive nanomedicines.^[^ ^4^ ^]^ Various pH‐ and glutathione‐responsive nanocarriers have been reported for antitumor drug delivery.^[^ ^5^ ^]^ Nevertheless, due to the limited difference of intracellular environment (acidic endosome/lysosome and high glutathione concentration) between tumor cells and normal cells, the selectivity of pH‐ and glutathione‐responsive nanocarriers is still limited.

Reactive oxygen species (ROS)‐responsive nanomedicines containing ROS‐sensitive chemical linkers (e.g., thioketal bond, peroxalate ester, diselenide bond) have attracted much attention in recent years.^[^ ^6^, ^15^ ^]^ An on‐demand drug release can be achieved by ROS‐induced cleavage of linkers and subsequent disintegration of nanocarriers. Due to the relatively low ROS level in normal cells, the ROS‐responsive nanomedicines have higher selectivity when compared to the commonly used pH‐ and glutathione‐responsive nanomedicines.^[^ ^7^ ^]^ Furthermore, ROS can also be used as a therapeutic agent to enhance the treatment efficacy of chemotherapy, achieving combination cancer therapy.^[^ ^8^ ^]^ Different strategies, such as photodynamic therapy (PDT), chemodynamic therapy, and sonodynamic therapy, have been developed for ROS generation.^[^ ^9^ ^]^ Among them, PDT, which employs light‐excited photosensitizer to generate reactive singlet oxygen (^1^O_2_) and destroy cancer cells, is a local therapy strategy with minimal damage to healthy tissues.^[^ ^10^ ^]^ The combination of PDT and ROS‐triggered drug release has been proven to have a potential for antitumor application.^[^ ^11^ ^]^

However, one limitation of ROS‐responsive nanocarriers is the serious consumption of ROS during the triggered drug release process. What's worse, the oxygen level at tumor site is usually insufficient;^[^ ^12^ ^]^ therefore, ROS consumption will be a major obstacle for achieving satisfactory combination treatment outcome. Herein, we propose a photodynamic‐chemodynamic cascade strategy for the design of drug delivery nanosystem. A doxorubicin hydrochloride (DOX)‐loaded ROS‐responsive polymersome (DOX‐RPS) is prepared via the self‐assembly of amphiphilic poly(ethylene glycol)‐poly(linoleic acid) (PEG‐PLA) and poly (ethylene glycol)‐(2‐(1‐hexyloxyethyl)‐2‐devinyl pyropheophorbide‐α)‐iron chelate (PEG‐HPPH‐Fe). As shown in **Figure** **1**, under physiological condition, the stable RPS can effectively deliver a drug to a tumor site through the passive targeting effect of nanomedicine. Once the DOX‐RPS is accumulated in the tumor tissue, upon laser irradiation, the photosensitizer HPPH will efficiently generate ROS, which further causes in situ oxidation of linoleic acid chain. The produced linoleic acid peroxide (LAP) will change the structural stability of RPS, permitting triggered drug release. Afterwards, catalyzed by HPPH‐Fe, ROS will be regenerated from LAP through a Fenton‐like reaction. Therefore, this photodynamic‐chemodynamic cascade strategy achieves ROS‐triggered drug release without serious ROS consumption and provides a promising approach for efficient drug delivery and enhanced combination therapy.

**Figure 1:**
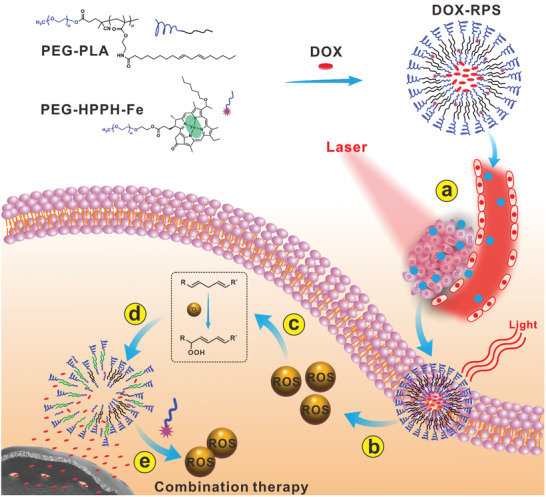
Schematic illustration of DOX‐RPS for drug delivery and chemo‐ROS combination therapy through photodynamic‐chemodynamic cascade reactions. a) The nanosized DOX‐RPS accumulates into tumor tissue though passive targeting; b) Upon laser irradiation, HPPH generates ROS through photodynamic reaction; c) The ROS further oxidizes linoleic acid to LAP; d) The produced LAP molecules change the structure of RPS, allowing drug release. e) In the presence of HPPH‐Fe, ROS are regenerated through Fenton‐like reaction. The produced ROS and released drug can achieve enhanced combination therapy.

The synthesis process of PEG‐PLA is shown in Figure S1 (Supporting Information). An amphiphilic polymer, poly(ethylene glycol)‐poly(2‐Boc‐amino)ethyl methacrylate (PEG‐PBOCAMA), was firstly synthesized through reversible addition fragmentation chain transfer polymerization. The degree of polymerization of PEG‐PBOCAMA determined by NMR was 24 and the calculated average molecular weight was 7.5 kDa (Figure S3, Supporting Information). Subsequently, the butyloxycarbonyl protection groups were removed by trifluoroacetic acid. Then linoleic acid was conjugated to polymer chain through the formation of amide bond, obtaining PEG‐PLA. The non‐responsive polymer, poly(ethylene glycol)‐poly(stearic acid) (PEG‐PSA), was also synthesized as a control. The photosensitizer‐containing polymer, poly(ethylene glycol)‐HPPH (PEG‐HPPH), was then synthesized as shown in Figure S2 (Supporting Information). The chemical structures of synthesized polymers were confirmed by NMR analysis (Figure S3–S7, Supporting Information). The PEG‐HPPH‐Fe was synthesized according to a previous report.^[^ ^13^ ^]^ Then, the DOX was encapsulated into polymersome self‐assembled from PEG‐PLA and PEG‐HPPH‐Fe, obtaining DOX‐RPS. Similarly, DOX‐loaded non‐responsive polymersome (DOX‐NRPS) was also prepared as a control by the assembly of PEG‐PSA and PEG‐HPPH‐Fe. The size distributions and morphologies of DOX‐RPS and DOX‐NRPS were determined by dynamic light scattering (DLS) and transmission electron microscopy (TEM), respectively. As shown in **Figure** **2**a and Figure S8 (Supporting Information), both DOX‐RPS and DOX‐NRPS had hydrodynamic diameters of about 80 nm and spherical vesicle structures. Moreover, the polymersomes exhibited good colloidal stabilities in phosphate buffered solution (PBS), which makes them suitable for in vivo applications (Figure 2b). The UV–Vis absorption spectra of DOX‐RPS and DOX‐NRPS showed typical peaks of DOX and HPPH, indicating the successful co‐loading of the drug and photosensitizer (Figure 2c).

**Figure 2:**
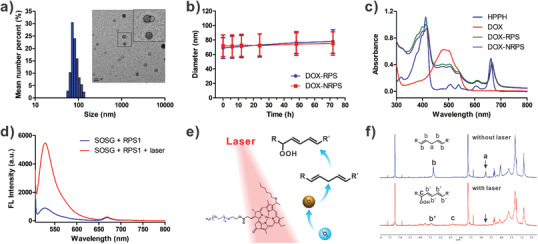
a) Particle diameter and TEM image (inset) of DOX‐RPS. b) Colloidal stabilities of DOX‐RPS and DOX‐NRPS (*n* = 3). c) Absorption spectra of HPPH, DOX, DOX‐RPS, and DOX‐NRPS. d) FL spectra of SOSG in the presence of RPS1 with or without laser irradiation. e) The mechanism of laser‐triggered generation of LAP. f) ^1^H NMR spectra of RPS1 with or without laser irradiation (671 nm, 100 mW cm^−2^, 20 min).

To study the ROS generation through cascade reactions, polymersomes without Fe (denoted as RPS1 and NRPS1, respectively) were prepared under the same experimental conditions except that the PEG‐HPPH‐Fe was replaced by PEG‐HPPH. In this way, the produced LAP will not undergo Fenton‐like reaction unless the catalyst iron(II) is added. Laser‐induced ^1^O_2_ generation of RPS1 was first evaluated by a fluorescence singlet oxygen sensor green (SOSG) method. As shown in the fluorescence (FL) spectra (Figure 2d), upon laser irradiation, the FL intensity of SOSG significantly increased, indicating a photo‐triggered ^1^O_2_ generation. Similar result was obtained in NRPS1 group (Figure S9, Supporting Information). It has been reported that linoleic acid could be oxidized by ^1^O_2_ to form LAP (Figure 2e).^[^ ^14^, ^16^ ^]^ To demonstrate the oxidation of linoleic acid chain and the generation of LAP under laser irradiation, the RPS1 suspensions with or without laser irradiation were lyophilized for NMR analysis. As shown in Figure 2f, the RPS1 without laser showed typical peaks of H‐a and H‐b at 2.75 and 5.2–5.5 ppm (attributed to linoleic acid). However, in the spectrum of laser‐irradiated RPS1, the peaks of H‐a and H‐b disappeared and peaks of H‐b′ at 5.3–6.2 ppm (attributed to conjugated double bonds), suggesting that linoleic acid chains were oxidized to LAP by ^1^O_2_.

The hydrodynamic size changes of DOX‐RPS and DOX‐NRPS with different times of laser irradiation were measured by DLS. As shown in Figure S10 (Supporting Information), upon laser irradiation, the hydrodynamic size of DOX‐RPS obviously increased, which might be due to the lipid peroxidation‐induced disassembly or expansion of DOX‐RPS. In contrast, the non‐responsive DOX‐NRPS didn't show hydrodynamic size change after 20 min of laser irradiation. The ROS‐triggered DOX‐release behaviors of DOX‐RPS were then investigated in vitro. The DOX‐RPS suspensions with different time durations of laser irradiation were dialyzed against PBS. As shown in **Figure** **3**a, DOX was released from the DOX‐RPS without laser irradiation at a relatively slow rate. However, the release of DOX was significantly accelerated under the treatment of laser irradiation. With the increase of laser irradiation time, the release rate of DOX obviously increased. The ROS‐triggered DOX release was likely due to the formation of hydrophilic peroxide groups in LAP, which would induce the permeability and structure stability changes of polymersome. In contrast, the drug release rates of DOX‐NRPS showed negligible differences no matter whether laser irradiation was applied or not (Figure 3b). These results demonstrated that the ^1^O_2_ generation and DOX release could be triggered by laser irradiation simultaneously.

**Figure 3:**
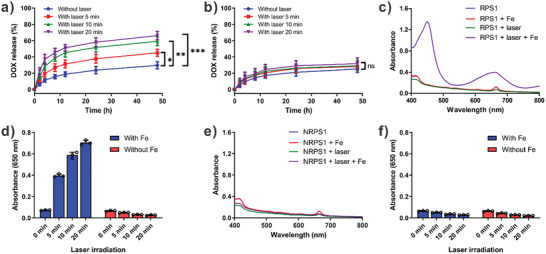
a) In vitro DOX release profiles of DOX‐RPS without or with different times of laser irradiation (*n* = 3, *p < 0.05, **p < 0.01, ***p < 0.001). b) In vitro DOX release profiles of DOX‐NRPS without or with different times of laser irradiation (*n* = 3). c) Absorption spectra of TMB in the presence of RPS1 or laser pre‐irradiated RPS1 (5 min) with or without Fe^2+^. d) Comparison of TMB oxidation by laser pre‐irradiated RPS1 (*n* = 3). e) Absorption spectra of TMB in the presence of NRPS1 or laser pre‐irradiated NRPS1 (5 min) with or without Fe^2+^. f) Comparison of TMB oxidation by laser pre‐irradiated NRPS1 (*n* = 3).

Because 3,3′,5,5′‐tetramethylbenzidine (TMB) molecules can be oxidized by the highly reactive radicals to give an absorbance change, the TMB assay was used to verify whether the ROS can be regenerated from LAP through Fenton‐like reaction. The RPS1 suspensions without or with laser pre‐irradiation were mixed with TMB solution. Then, the absorption spectra were measured in the presence or absence of catalyst iron(II). As shown in Figure 3c, without laser irradiation, neither RPS1 group nor RPS1 plus iron(II) group can lead to an absorbance increase of TMB. However, for the laser pre‐irradiated RPS1, the absorbance of TMB was obviously increased in the presence of iron(II), indicating oxidation of TMB. In contrast, no obvious absorbance change was observed without adding iron(II) (Figure 3d). As a non‐responsive control, NRPS1 could not oxidize TMB under all conditions (Figure 3e,f). These results confirmed that the LAP could regenerate ROS through iron(II) catalyzed Fenton‐like reaction.

The intracellular ROS generation was then investigated on U87MG cells by using an ROS probe, 2′,7′‐dichlorofluorescin diacetate (DCFDA), which can be oxidized by ROS to produce dichlorofluorescein (DCF) with green fluorescence. As shown in the flow cytometry (FCM) analysis results (**Figure** **4**a), in control group, laser irradiation alone didn't affect the fluorescence intensity; however, with the incubation of RPS1 and subsequent laser irradiation, the DCF fluorescence intensity inside cells was obviously enhanced. The NRPS1‐treated cells showed similar results to those treated with RPS1 (Figure S11, Supporting Information). These results indicated light‐induced ^1^O_2_ generation capabilities of the RPS1 and NRPS1. Then the cells were incubated with DCFDA and RPS1 or laser pre‐irradiated RPS1. The FCM results showed that laser pre‐irradiated RPS1 could lead to fluorescence intensity enhancement when compared to RPS1 (Figure 4b), indicating oxidation of DCFDA by LAP. In contrast, negligible differences were observed between NRPS1 and laser pre‐irradiated NRPS1 (Figure S12, Supporting Information). Furthermore, the light‐controlled drug distribution was investigated on U87MG cells. Fluorescent images were acquired by confocal laser scanning microscopy after nuclear staining with 4′,6‐diamidino‐2‐phenylindole (DAPI). As shown in Figure 4c, strong red fluorescence inside cell nucleus could be observed in free DOX groups with or without laser irradiation, indicating strong ability of free DOX to enter cells and diffuse into nucleus. Remarkably, in the DOX‐RPS incubated cells, laser irradiation showed an obvious influence on drug distribution. Without the laser, the DOX fluorescence mainly distributed in cytoplasm; however, red fluorescence could be detected in both the cytoplasm and nucleus when the laser irradiation was applied. These results demonstrated that the drug release of DOX‐RPS could be accelerated by laser irradiation. In contrast, for the cells treated with DOX‐NRPS, negligible fluorescence was detected in the nuclei even if laser irradiation was applied, indicating the non‐responsiveness of the NRPS.

**Figure 4:**
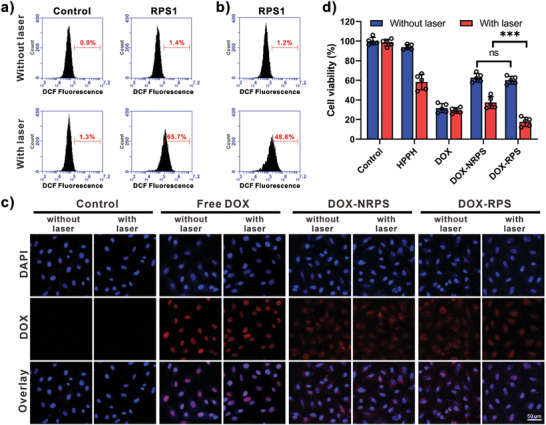
a) FCM analysis of U87MG cells incubated with DCFDA and RPS1, followed by laser irradiation (100 mW cm^−2^, 5 min). b) FCM analysis of U87MG cells incubated with DCFH‐DA and laser pre‐irradiated RPS1. c) Confocal fluorescence images of different samples treated U87MG cells with or without laser irradiation (100 mW cm^−2^, 5 min). Nuclei were stained with DAPI (blue). d) Relative viability of different samples treated U87MG cells (HPPH concentration: 0.05 µm, DOX concentration: 0.25 µm, with or without laser irradiation) for 48 h (*n* = 5, ***p < 0.001).

The cytotoxicity of drug free RPS was evaluated on U87MG, A549, and 293T cells by methyl thiazolyl tetrazolium (MTT) assay. As shown in Figure S13 (Supporting Information), a negligible cytotoxicity was observed even at high concentration. Then the anticancer activities of free HPPH, free DOX, and nanomedicines with or without laser irradiation were evaluated on U87MG cells by MTT assay. The viabilities of cells treated with free HPPH or free DOX at various concentrations were measured. As shown in Figure S14 (Supporting Information), HPPH without laser irradiation exhibited weak cytotoxicity even at a concentration of 2 µm; however, laser irradiation significantly enhanced the cytotoxicity of HPPH. The IC_50_ value of HPPH under laser irradiation was determined to be 58.3 nm, indicating the efficient PDT effect of HPPH. As a chemotherapeutic drug, free DOX showed comparable cytotoxicities in both conditions (Figure S15, Supporting Information). The IC_50_ values of free DOX with or without laser irradiation were determined to be 89.1 and 92.9 nm, respectively. Then the anticancer activities of different groups (free HPPH, free DOX, DOX‐NRPS, and DOX‐RPS) were measured at the same concentrations of HPPH and DOX. As shown in Figure 4d, the DOX‐RPS and DOX‐NRPS without irradiation exhibited comparable cell cytotoxicities, which were attributed to the slow DOX release from the polymersomes. Under laser irradiation, an obvious cell viability decrease was observed in nanomedicine groups, attributing to the chemo‐PDT combination effect. It is noteworthy that the DOX‐RPS exhibited more potent anticancer activity when compared to the DOX‐NRPS, which was attributed to the ROS‐accelerated drug release and ROS regeneration.

Then we investigated the in vivo performance of DOX‐RPS on U87MG‐tumor‐bearing mice. Positron emission tomography (PET) imaging was used to study the tumor accumulation and biodistribution of DOX‐RPS. To chelate with the radionuclide zirconium‐89 (^89^Zr), deferoxamine was conjugated to the DOX‐RPS. The obtained ^89^Zr‐labeled DOX‐RPS was administered by intravenous injection. At different time points (1, 4, 24, 48, and 72 h) postinjection, the decay‐correlated PET images of mice were acquired. As shown in **Figure** **5**a, the DOX‐RPS was mainly distributed in heart and blood circulation within 4 h after injection. An obvious signal could be observed in the heart area even at 24 h postinjection, indicating great in vivo stability and long blood circulation of the DOX‐RPS. A tumor signal could be observed from 4 h postinjection and the tumor signal intensity gradually increased with time, attributing to the tumor accumulation through passive targeting. The region‐of‐interest (ROI) analysis results (Figure 5b,c) showed that the concentration of DOX‐RPS in heart (with blood) decreased over time; while liver and tumor uptakes increased. At 24 h postinjection, a remarkable tumor uptake of 5.72%ID g^−1^ was achieved. The maximum value of tumor uptake was 6.87%ID g^−1^ at 48 h postinjection. The mice were sacrificed at 72 h postinjection for ex vivo biodistribution study. Tumors and major organs (heart, liver, spleen, lung, and kidneys) were collected and the levels of radioactivity in these tissues were quantified by using a *γ*‐counter (Figure 5d). The results showed that the DOX‐RPS was mainly accumulated in tumor, liver, and spleen, which were consistent with PET images and ROI analysis results. In general, the in vivo performance of nanoparticles is mainly affected by composition, particle size, and surface properties. Considering the similar size, morphology and surface property of DOX‐RPS and DOX‐NRPS, the DOX‐NRPS is expected to show similar in vivo behavior with the DOX‐RPS.

**Figure 5:**
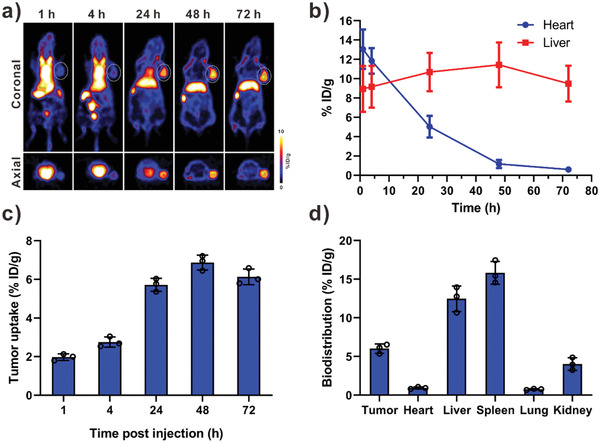
a) PET images of U87MG tumor‐bearing mice after intravenous injection of ^89^Zr labeled DOX‐RPS. b) The distribution of ^89^Zr‐labeled DOX‐RPS in heart (with blood) and liver at different time points after injection. c) Tumor uptake of ^89^Zr labeled DOX‐RPS at 1, 4, 24, 48, and 72 h post‐injection. d) Biodistribution of tumor and primary organs at 72 h postinjection. (*n* = 3)

Encouraged by the potent anticancer activity in vitro and high tumor accumulation of the DOX‐RPS in vivo, we further evaluated the in vivo chemo‐photodynamic combination therapy. Different samples including saline, free DOX, RPS, DOX‐RPS, and DOX‐NRPS (DOX‐equivalent dose: 5 mg kg^−1^) were intravenously injected into U87MG tumor‐bearing mice every 3 days for 2 times. Based on the in vivo PET imaging results, an effective tumor accumulation was achieved at 24 h postinjection; therefore, laser irradiation (100 mW cm^−2^, 10 min) was applied in the tumor sites of certain groups at 24 h postinjection. As shown in the tumor growth curves (**Figure** **6**a), compared with saline control group in which tumors grew rapidly, all the treatment groups showed inhibition on tumor growth. On the 14th day, the inhibition rate of tumor growth (IRG) for PDT only (RPS + laser) group and chemotherapy only (DOX‐RPS) group were calculated to be 43.2% and 56.8%, respectively (Figure 6b). It is noteworthy that the DOX‐RPS + laser group exhibited the most potent antitumor effect with an IRG of 89.1%, which was higher than that of DOX‐NRPS + laser group (73.1%). The treatment‐induced tumor cell apoptosis was further confirmed by hematoxylin and eosin (H&E) staining of tumor tissue sections (Figure 6e). The efficacious therapeutic effect of DOX‐RPS + laser group was likely due to the ROS‐triggered drug release and ROS regeneration. As a treatment outcome, the survival time of DOX‐RPS‐ and laser‐treated mice was greatly prolonged (Figure 6c). Furthermore, due to the systemic distribution of highly toxic chemotherapeutic drugs, obvious mice body weight loss caused by administration of free DOX was observed in the first 4 days, indicating serious side effect (Figure 6d). Afterwards, the weight of the free DOX‐treated mice gradually recovered and increased due to the fast metabolism of the injected free DOX. In contrast, the treatment of nanomedicines did not cause obvious body weight loss.

**Figure 6:**
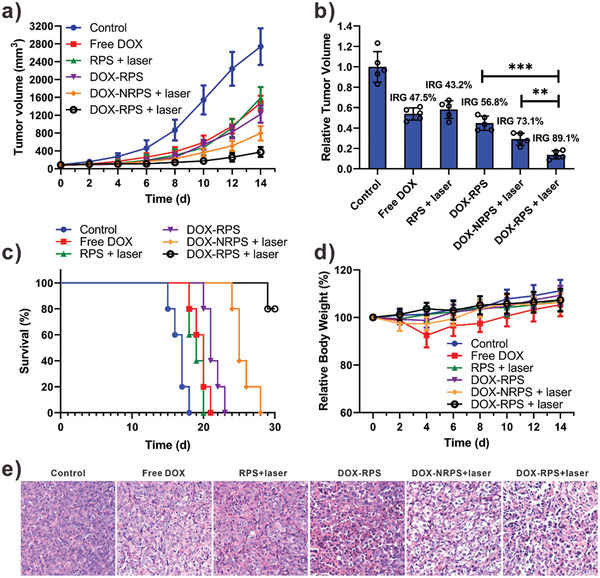
a) Tumor growth curves of the mice upon different treatments (laser: 100 mW cm^−2^, 10 min). b) The relative tumor volume and inhibition rate of tumor growth (IRG) at the end of treatment (*n* = 5, **p < 0.01, ***p < 0.001). c) Survival curves of the mice. d) Mice body‐weight changes during the treatments. e) H&E analyses of tumor tissues after different treatments.

In summary, a DOX and HPPH co‐loaded ROS‐responsive polymersome was developed for chemo‐photodynamic combination cancer therapy. The DOX‐RPS can effectively accumulate in tumor tissue through passive targeting effect and respond to laser irradiation applied at tumor site. The ROS generated by photodynamic effect would oxidize the linoleic acid to LAP and cause permeability and structure stability changes of polymersome, resulting in ROS‐triggered drug release. Furthermore, ROS could be regenerated from LAP through a HPPH‐Fe catalyzed Fenton‐like reaction. Therefore, excessive ROS consumption in the ROS‐triggered drug release process was avoided through the photodynamic‐chemodynamic cascade reactions. The ROS generation and regeneration, triggered drug release behavior, and potent anticancer effect of the DOX‐RPS were confirmed by in vitro and in vivo results. This study provides a promising strategy for the design of nanomedicines to achieve enhanced chemo‐photodynamic combination therapy.

## Experimental Section

The experimental section is available in the Supporting Information.

All animal experiments were performed under a National Institutes of Health Animal Care and Use Committee (NIHACUC) approved protocol.

## Conflict of Interest

The authors declare no conflict of interest.

## Supporting information

Supporting Information
