# Supplementary material for: Photodynamic‐Chemodynamic Cascade Reactions for Efficient Drug Delivery and Enhanced Combination Therapy
Source: Adv Sci (Weinh). 2021 Apr 8;8(10):2002927. doi: 10.1002/advs.202002927 (PMC8132047; doi:10.1002/advs.202002927)
Supplement: Supplementary file 1 — Supporting Information [file ADVS-8-2002927-s001.pdf]

© 2020 Wiley-VCH GmbH

ADVANCED SCIENCE Open Access

Supporting Information

for *Adv. Sci.*, DOI: 10.1002/advs.202002927

Photodynamic-Chemodynamic Cascade Reactions for Efficient Drug Delivery and Enhanced Combination Therapy

Sheng Wang, Guocan Yu, Weijing Yang, Zhantong Wang, Orit Jacobson, Rui Tian, Hongzhang Deng, Lisen Lin* and Xiaoyuan Chen**

Supporting Information

Photodynamic-Chemodynamic Cascade Reactions for Efficient Drug Delivery and Enhanced Combination Therapy

Sheng Wang, Guocan Yu, Weijing Yang, Zhantong Wang, Orit Jacobson, Rui Tian, Hongzhang Deng, Lisen Lin* and Xiaoyuan Chen**

Materials

Poly(ethylene glycol) methyl ether (4-cyano-4-pentanoate dodecyl trithiocarbonate) (PEG-RAFT, average Mn 2400), Poly(ethylene glycol) methyl ether (PEG-OH, average Mn 2000), (2-Boc-amino) ethyl methacrylate (BOCAMA), 2,2′-azobis(2-methylpropionitrile) (AIBN), trifluoroacetic acid (TFA), 2′,7′-dichlorofluorescin diacetate (DCFH-DA), methyl thiazolyl tetrazolium (MTT), 4′,6-diamidino-2-phenylindole (DAPI) were obtained from Sigma-Aldrich. Linoleic acid (LA), stearic acid (SA), 1-(3-dimethylaminopropyl)-3-ethylcarbodiimide hydrochloride (EDC), N-hydroxysuccinimide (NHS), 4-dimethylaminopyridine (DMAP), triethylamine (TEA), 3,3',5,5'-tetramethylbenzidine (TMB) were purchased from TCI. HPPH was purchased from MedChemExpress LLC.

Synthesis of PEG-PLA and PEG-PSA

The PEG-PBOCAMA was first synthesized by a Reversible Addition–Fragmentation chain Transfer (RAFT) polymerization method (Figure S1). Briefly, PEG-RAFT agent (0.04 mmol), BOCAMA (1 mmol), AIBN (0.008 mmol) were dissolved in 1, 4-dioxane (3 mL) and added into a flask. The flask was sealed under dry argon and the polymerization was carried out at 70 °C for 24 h. The mixture was precipitated into an excess of hexane to generate PEG-PBOCAMA. Then the BOC protection groups of PEG-PBOCAMA were removed by TFA, obtaining PEG-PAMA. Afterwards, LA (32 mg) was dissolved in THF (6 mL) under dry argon; EDC (30 mg) and NHS (20 mg) were dissolved in DMSO (2 mL) and added into the

LA solution for activation. Subsequently, PEG-PAMA (40 mg) and TEA (30 μL) were dissolved in THF (4 mL) and added into the mixture. The reaction was carried out at room temperature for 24 h. The product was dialyzed in DMSO and pure water and then lyophilized to generate PEG-PLA. The PEG-PSA was synthesized under the same experimental conditions except that the LA was replaced with SA.

Synthesis of PEG-HPPH-Fe

The PEG-HPPH was first synthesized (Figure S2). HPPH (15 mg), EDC (10 mg), DMAP (0.5 mg) were dissloved in dichloromethane (10 mL). PEG-OH (90 mg) was added into the mixture for reaction overnight at room temperature. Then the dichloromethane was removed by reduced pressure distillation. The product was dialyzed in pure water and then lyophilized to generate PEG-HPPH. The PEG-HPPH-Fe was synthesized according to a previous report.[1] PEG-HPPH was incubated with 10 fold excess free iron(II) acetate in methanol for 1 hour at room temperature under argon. Free metal was removed by dialysis. The PEG-HPPH-Fe was then lyophilized.

Characterizations of polymers

^{1}H NMR spectra were recorded on a JEOLGX 400 D spectrometer (400 MHz) with $CDCl_3$ as the solvent and tetramethyl silane as an internal standard. The degree of polymerization of PEG-PBOCAMA determined by NMR is ~24.

Preparation and characterizations of DOX-RPS and DOX-NRPS

The DOX-RPS was prepared as follows: PEG-PLA and PEG-HPPH-Fe were dissolved in 4 mL of dichloromethane at room temperature to obtain the organic phase. DOX hydrochloride was dissolved in 0.5 mL of pure water and mixed with the organic phase under sonication. The obtained emulsion was added to 8 mL of pure water and sonicated for another 120 s. The organic solvents were evaporated on a rotary evaporator to form DOX-RPS suspension. The DOX-NRPS was prepared under the same experimental conditions except that the PEG-PLA was replaced with PEG-PSA. RPS and NRPS without drug loading were also prepared. The

morphology of the RPS and NRPS was observed by Tecnai TF30 transmission electron microscope (TEM) (FEI, Hillsboro, OR). The effective particle diameters and zeta potential of the samples were determined by a SZ-100 nano particle analyzer (HORIBA Scientific, USA) at room temperature. UV-vis absorption spectra of the samples were measured by Genesys 10S UV-Vis spectrophotometer (Thermo Scientific, Waltham, MA).

ROS generation

In order to study the ROS generation through photodynamic and chemodynamic reactions, polymersomes without Fe (denoted as RPS1 and NRPS1) were prepared under the above-mentioned experimental conditions except that the PEG-HPPH-Fe was replaced with PEG-HPPH. The singlet oxygen generation of RPS1 under laser irradiation was evaluated by a fluorescence singlet oxygen sensor green (SOSG) method. Briefly, SOSG was mixed with RPS1 suspension, the final SOSG oncentration was adjusted to 2 μM. The fluorescence spectra of the mixture solution before and after laser irradiation (671 nm) were measured under excitation at 498 nm. The generation of ROS through Fenton-like reaction was determined by a TMB assay. The RPS1 suspensions without or with 671 nm laser pre-irradiation (100 mW cm^{-2}, 5 min) were mixed with TMB solution. Absorption spectra were measured in the presence or absence of catalytic Fe^{2+} ions.

***In vitro* drug release**

The *in vitro* DOX release behaviors of the samples in different conditions were evaluated at 37 °C ($n = 3$). The samples without or with 671 nm laser irradiation were dispered in media (2 mL) and added to dialysis bags (MWCO: 3500 Da) and placed in environmental media (20 mL). At appropriate time points, 2 mL of the medium was taken out and replaced with the same amount of fresh medium. The amount of the released DOX was measured by UV-Vis spectrophotometer at the wavelength of 480 nm.

***In vitro* cell experiments**

The U87MG, A549 and 293T cell lines were purchased from American type culture collection (ATCC). For cytotoxicity study, cells were seeded into 96-well plates at a density of 3×10^3 cells per well ($n = 5$) and incubated with different concentrations of RPS for 48 h. The relative cell viabilities were measured by MTT assay.

To assess the intracellular ROS generation, U87MG cells were seeded into 8-well plates and incubated with DCFH-DA (15 μM) and different samples (RPS1, NRPS1, laser pre-irradiated RPS1 and laser pre-irradiated NRPS1) for 2 h. Then the intracellular ROS level was determined by flow cytometry (FCM) analyse.

To assess the cellular uptake of samples, U87MG cells were seeded into 8-well plates. Different samples (DOX concentration: 10 mg L^{-1}) without or with 671 nm laser pre-irradiation (100 mW cm^{-2}, 5 min) were added into wells. Then the cells were incubated with samples for 4 h. The culture media were removed, and the cells were fixed with Z-Fix solution and stained with DAPI. The cellular uptake and drug distribution were determined by confocal laser scanning microscopy (CLSM).

For *in vitro* antitumor activities, U87MG cells were seeded into 96-well plates at a density of 3×10^3 cells per well ($n = 5$) and incubated with different samples (HPPH concentration: 0.05 μM, DOX concentration: 0.25 μM) for 8 h, followed by exposure to a 671 nm laser (100 mW cm^{-2}, 5 min). Then the cells were incubated for another 40 h. Thereafter, the relative cell viabilities were measured by MTT assay.

Animal model

All animal experiments were performed under a National Institutes of Health Animal Care and Use Committee (NIHACUC) approved protocol. Athymic nude mice (Harlan, Indianapolis, IN) were subcutaneously implanted with 3×10^6 of U87MG cells.

***In vivo* PET imaging**

Deferoxamine (DFO) conjugated polymer was synthesized by reaction between SCN-DFO and excessive amino groups of PEG-PAMA. Then the DFO-PEG-PLA was synthesized by

the above-mentioned method. DFO-modified DOX-RPS was prepared to chelate with the radionuclide zirconium-89 (^{89}Zr).[2] The ^{89}Zr-DOX-RPS solution (100 μL, 200 μCi) was intravenously injected into U87MG tumor-bearing mice. An Inveon small-animal PET scanner (Siemens, Erlangen, Germany) was used for the scanning at indicated time points after injection. At 72 h post-injection, the mice were sacrificed and the major organs were collected and assayed for radioactivity using a gamma counter. The percent of injected dose/gram of tissue (%ID/g) was then calculated (n = 3).

***In vivo* therapy**

U87MG tumor-bearing mice were randomly divided into 5 groups: control group, free DOX group, RPS + laser group, DOX-RPS group and DOX-RPS + laser group. When the tumors reached about 60 mm^3, the mice were treated with samples (5 mg DOX kg^{-1}) via intravenous injection every 3 days for 2 times. A 671 nm laser irradiation (100 mW cm^{-2}, 10 min) was applied in the laser irradiation groups at 24 h postinjection. Tumor size and body weight were monitored every 2 days. Tumor volume (V) was calculated as (major axis) × (minor axis)^2/2. On the 14th day, the relative tumor volume for each experimental group was calculated as V_{exp} / $V_{control}$; the inhibition rate of tumor growth (%) was calculated as {1 - [($V_{expday14}$ - $V_{expday0}$) / ($V_{controlday14}$ - $V_{controlday0}$)]} × 100. Mice were euthanized when major axis of tumor exceeded 20 mm or when mouse weight lost by over 20%. After treatment, one mouse of each group were euthanized, major organs and tumors were collected for hematoxylin and eosin (H&E) staining.[3] H&E staining slides were prepared by BBC Biochemical (Mount Vernon, WA) and observed using a BX41 bright field microscopy (Olympus).

[1] J. F. Lovell, C. S. Jin, E. Huynh, H. Jin, C. Kim, J. L. Rubinstein, W. C. Chan, W. Cao, L. V. Wang, G. Zheng, *Nat. Mater.* **2011**, *10*, 324-332.
[2] S. Wang, G. Yu, Z. Wang, O. Jacobson, L. S. Lin, W. Yang, H. Deng, Z. He, Y. Liu, Z.-Y. Chen, X. Chen, *Angew. Chem. Int. Ed.* **2019**, *58*, 14758-14763.
[3] W. Tang, Z. Yang, S. Wang, Z. Wang, J. Song, G. Yu, W. Fan, Y. Dai, J. Wang, L. Shan, G. Niu, Q. Fan, X. Chen, *ACS Nano* **2018**, *12*, 2610-2622.

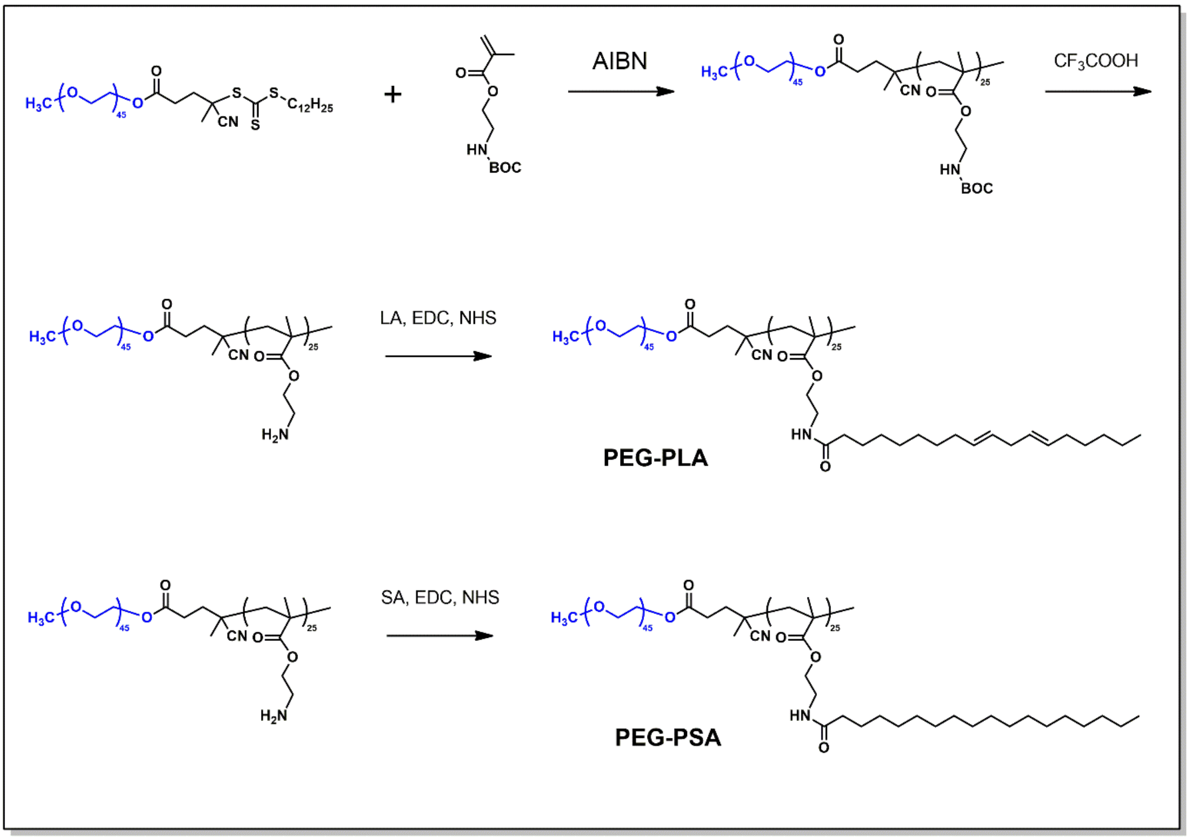

Figure S1. Synthesis processes of PEG-PLA and PEG-PSA.

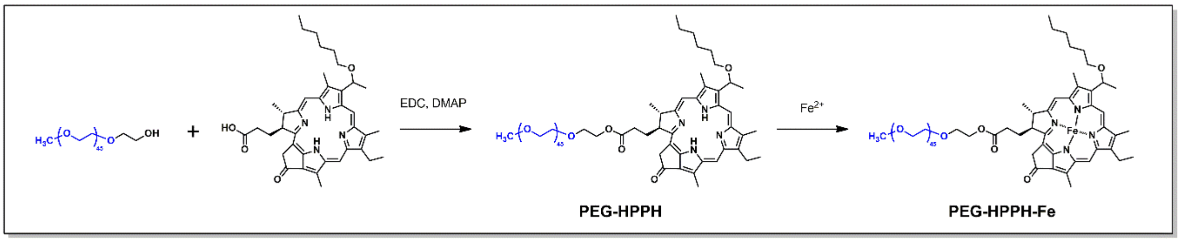

Figure S2. Synthesis process of PEG-HPPH-Fe.

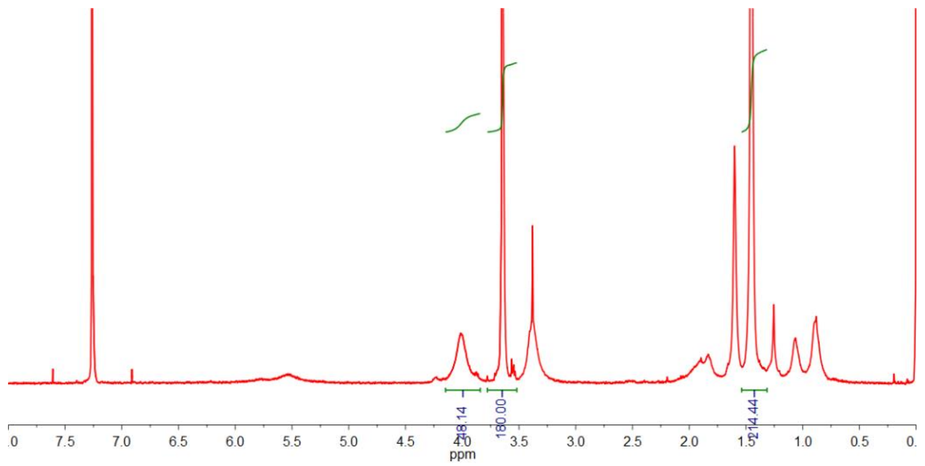

Figure S3. ^{1}H NMR spectrum of PEG-PBOCAMA.

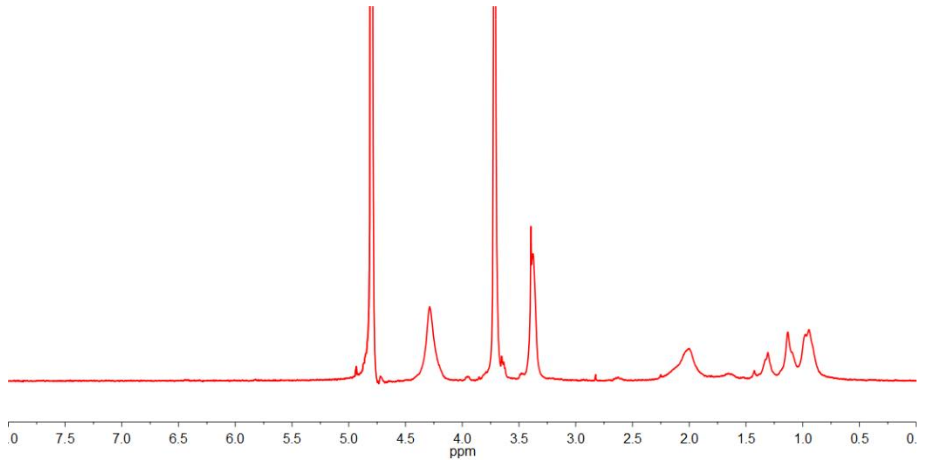

Figure S4. ^{1}H NMR spectrum of PEG-PAMA.

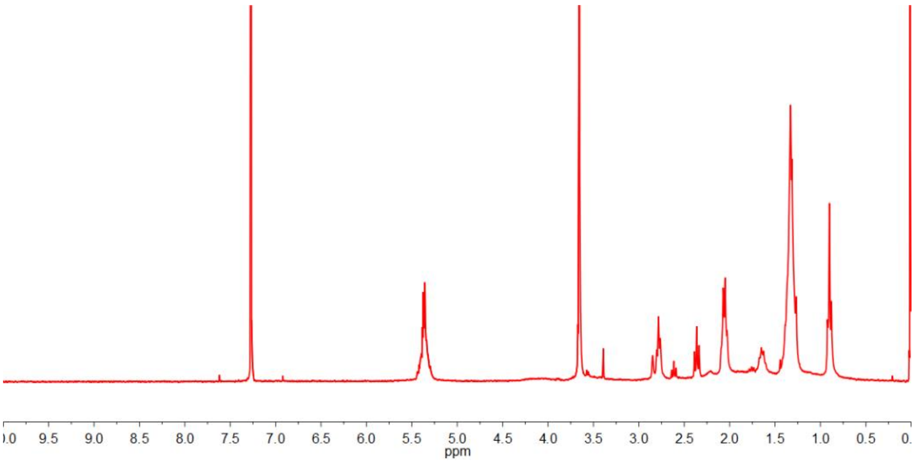

Figure S5. ^{1}H NMR spectrum of PEG-PLA.

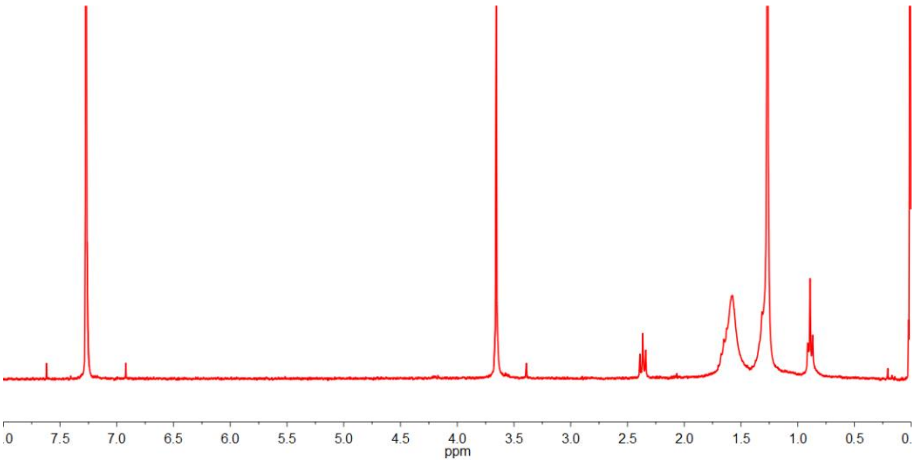

Figure S6. ^{1}H NMR spectrum of PEG-PSA.

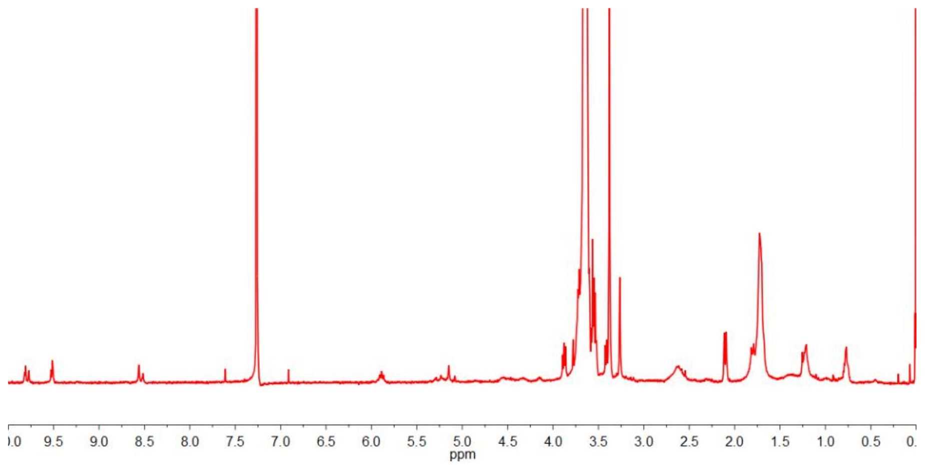

Figure S7. ^{1}H NMR spectrum of PEG-HPPH.

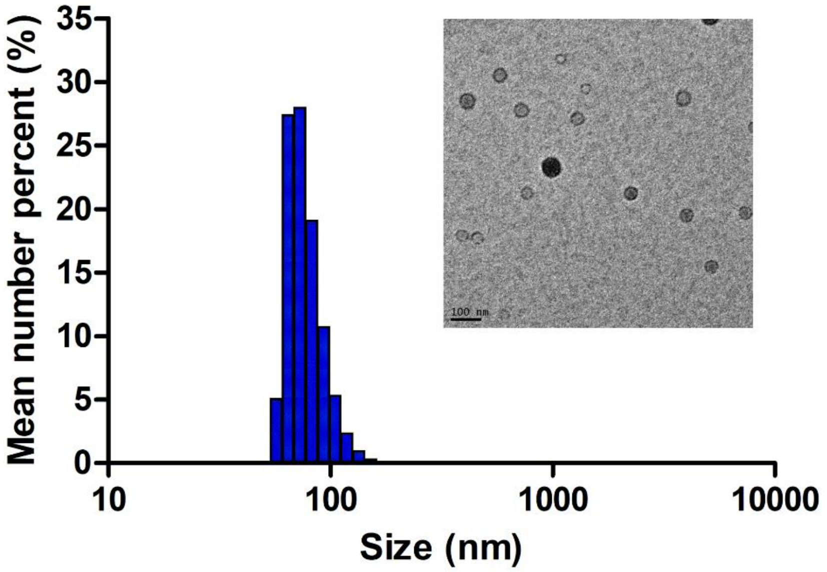

Figure S8. Particle diameter and TEM image of DOX-NRPS.

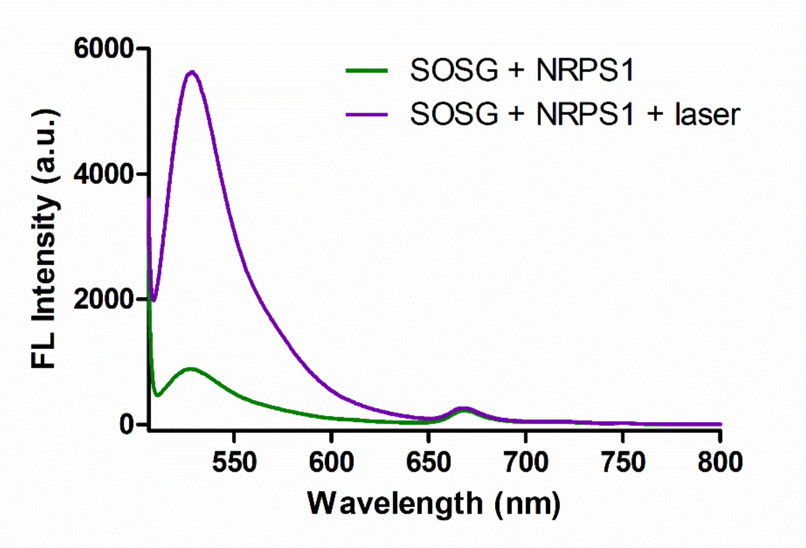

Figure S9. FL spectra of SOSG in the presence of NRPS1 with or without laser irradiation.

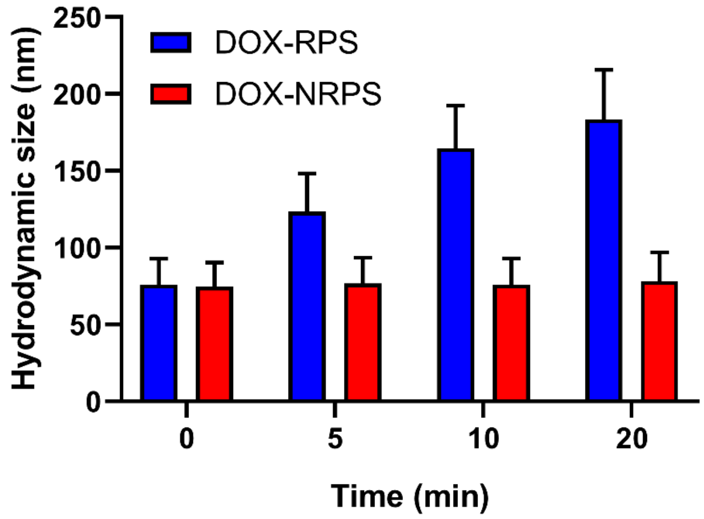

Figure S10. Hydrodynamic size changes of DOX-RPS and DOX-NRPS with different times of laser irradiation.

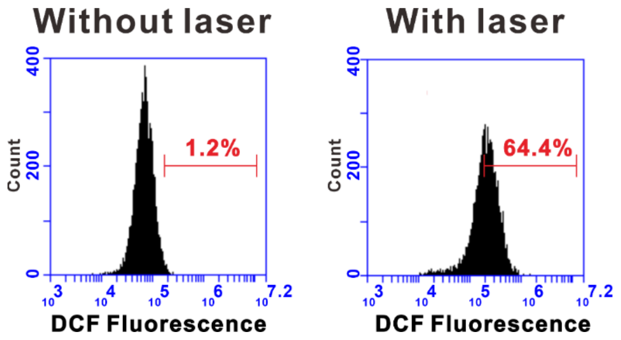

Figure S11. FCM analysis of U87MG cells incubated with DCFH-DA and NRPS1. Laser irradiation was performed after incubation (671 nm, 100 mW cm^{-2}, 5 min).

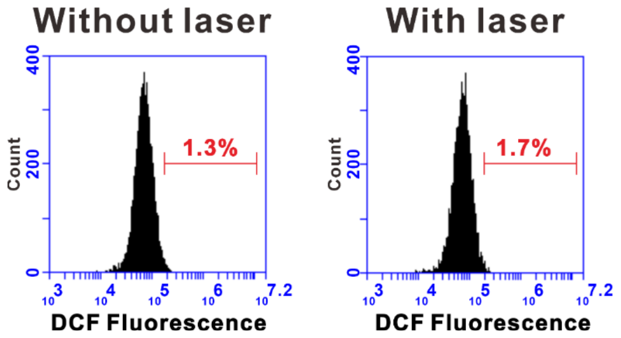

Figure S12. FCM analysis of U87MG cells incubated with DCFH-DA and laser pre-irradiated NRPS1 (671 nm, 100 mW cm^{-2}, 5 min).

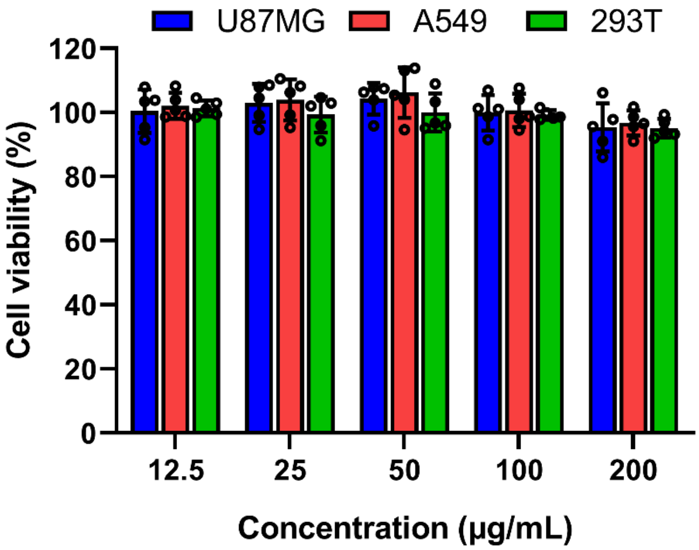

Figure S13. Relative viability of U87MG, A549 and 293T cells incubated with RPS for 48 h (n = 5).

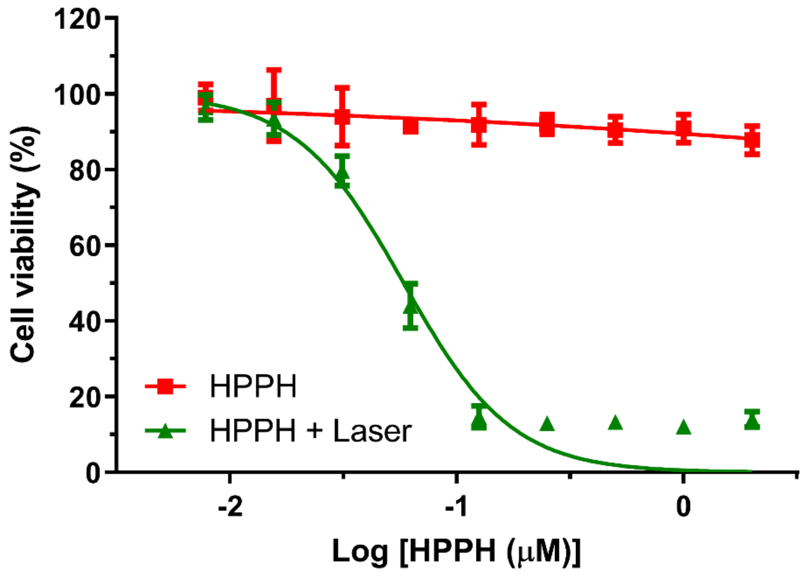

Figure S14. Relative viability of U87MG cells incubated with HPPH for 48 h with or without laser irradiation (671 nm, 100 mW cm^{-2}, 5 min).

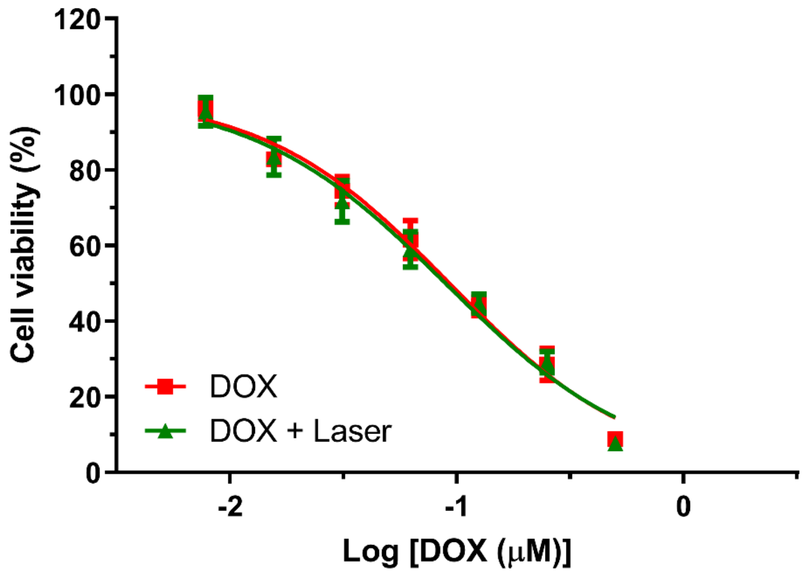

Figure S15. Relative viability of U87MG cells incubated with DOX for 48 h with or without laser irradiation (671 nm, 100 mW cm^{-2}, 5 min).
